# Association of Chronic Hepatitis B With Colorectal Cancer and Its Dual Impact on Colorectal Liver Metastasis: A Narrative Review

**DOI:** 10.7759/cureus.76079

**Published:** 2024-12-20

**Authors:** Avneet Kaur, Gibran A Azeez, Mounika Thirunagari, Nazeefa Fatima, Abhinav Anand, Aadi R Palvia, Ann Kashmer Yu

**Affiliations:** 1 Internal Medicine, California Institute of Behavioral Neurosciences & Psychology, Fairfield, USA

**Keywords:** adenoma carcinoma sequence, chronic hepatitis b, colorectal adenoma, colorectal cancer, colorectal liver metastasis, hepatitis b virus, screening colonoscopy, tumor suppressor gene (tp53) mutation

## Abstract

Viral hepatitis B is infamous for being contracted in young adulthood and adolescence, as high-risk behaviors like unprotected sexual intercourse and intravenous drug abuse are common. Most infections caused by the hepatitis B virus (HBV) are cleared without any long-term sequelae, but some may persist and cause chronic hepatitis B (CHB). This chronicity may produce a state of prolonged inflammation and significantly increase the risk of developing colorectal adenomas (CRA) and colorectal carcinomas (CRC). The aim of this review is to deep-dive into the mechanisms by which CHB may predispose a patient to develop CRA and, more grimly, CRC. It also focuses on studying the influence of CHB on colorectal cancer liver metastases (CRLM). We conducted a comprehensive literature search using databases like PubMed and Google Scholar, focusing on studies that investigate the role of HBV in colorectal carcinogenesis and CRLM rates in patients suffering from CHB. Chronic inflammation, viral protein interactions with tumor suppressor genes, alteration of cellular pathways such as wingless-related integration site (Wnt) signaling, and extrahepatic accumulation of hepatitis B surface antigen (HBsAg) were the key mechanisms identified. Quite peculiarly, CHB, which is thought to increase the risk for CRA, seemed to protect against CRLM probably due to its sclerosing effect on the liver parenchyma and due to certain immune-mediated mechanisms that suppress tumor growth. Nonetheless, high viral count or the presence of hepatitis B envelope antigen (HBeAg) was found to increase the risk for CRLM, potentially due to increased angiogenesis in the liver. These findings provide convincing evidence that enhanced colonoscopic screening and stronger management protocols for patients suffering from it have the potential to reduce the risk of developing CRC and CRLM.

## Introduction and background

Chronic viral hepatitis caused by both hepatitis B virus (HBV) and hepatitis C virus (HCV) is of great public health concern on a global scale [1]. It is well established that around ten percent of patients who suffer from hepatitis B infection may progress to chronicity, which has a poor prognosis in terms of morbidity and mortality. In the United States, over 2 million people live with chronic hepatitis B (CHB). It is more prevalent in African Americans and Asians as compared to their Caucasian counterparts [2,3]. Transmission usually occurs through sexual activity and contact with infectious bodily fluids rather than the fecal-oral route. HBV is a deoxyribonucleic acid (DNA) virus that can directly integrate into the host genome. This then leads to the production of transforming proteins like protein X and preS-S [4]. Hepatic sequelae of CHB are well known, but its association with extrahepatic malignancies, including but not limited to pancreatic cancer, non-Hodgkin lymphomas, chronic lymphocytic leukemia, and other solid organ tumors, is poorly demonstrated [5-9]. Colorectal carcinoma (CRC) is a matter of grave concern as it is the third leading cause of cancer-related deaths [10,11]. Some noteworthy risk factors for the development of CRC are obesity, consumption of red meat, low-fiber diet, positive family history of CRC, lack of exercise, and inflammatory bowel disease [12]. Although previous literature has studied the association between hepatitis C infection and colon adenomas, with the HCV envelope protein E2 stimulating B cell growth and the core protein inhibiting tumor protein p53 (TP53) and activating nuclear factor-kappa beta (NF-κB), resulting in tumor growth, similar studies regarding the relationship between chronic hepatitis B and colorectal neoplasia have yielded inconclusive results [13-15]. By the purpose of this review, we aim to comprehensively understand all the possible underlying pathophysiological mechanisms occurring in CHB patients that can be contributing to their elevated risk of developing carcinogenesis in the colon and to determine if CHB can be an independent risk factor for colon cancer. Knowing the influence of various stages of CHB on the rates of CRLM can also be helpful in predicting the prognosis and survival of these patients. This can prompt healthcare providers to implement a more rigorous and early colon cancer screening protocol and form management strategies according to individual patient risk factors for developing CRC. Also, it can be helpful to better educate the patients by emphasizing the importance of preventive strategies, including lifestyle modifications and hepatitis B vaccinations, to mitigate their elevated risk of developing CRC. This review also provides a foundation for the future development of novel therapeutic agents that can target both of these conditions simultaneously.

## Review

Methods

A thorough literature search was conducted across two major databases, PubMed and Google Scholar, using specific keywords related to colorectal cancer and chronic hepatitis B, emphasizing the disease's carcinogenesis and metastasis. The search results contained keywords such as "chronic hepatitis B," "colorectal adenoma," "colorectal carcinoma," "colorectal carcinogenesis," and "colorectal liver metastasis," which were applied alone and in combination to maximize the scope of our review.

Our inclusion criteria involved a prime focus on the studies that looked at the connection between chronic hepatitis B infection and colorectal cancer (CRC) or colorectal liver metastases (CRLM). We specifically sought articles that explored the mechanisms through which chronic hepatitis B may drive colorectal carcinogenesis or enhance its metastatic potential. To ensure that historical and modern perspectives were represented, we included articles published in the broader period from 1994 to 2024. No restrictions were applied regarding study design, publication type, or language, ensuring that the selection process remained thorough. Therefore, population-based research, case-control studies, retrospective analyses, and systematic reviews were eligible. All identified articles underwent a two-step screening process: an initial review of titles and abstracts followed by a thorough full-text analysis to verify their applicability to our study objectives. Duplicate records and studies not meeting the focus of our theme were excluded from further consideration.

All authors worked collaboratively and followed a standardized selection process to ensure methodological rigor. The authors independently evaluated each article, and any discrepancies in study inclusion were resolved through consensus discussions. In the end, 16 papers were chosen to be the focus of our review. These studies were analyzed in detail, and their findings were synthesized to highlight the key mechanisms and the impact of chronic hepatitis B infection on colorectal carcinogenesis and liver metastasis.

Results

We reviewed 16 studies that examined the connection between colorectal neoplasia, chronic hepatitis B (CHB), and colorectal liver metastases (CRLM) between 1994 and 2024. Many studies found that HBV infection was strongly associated with an increased risk of colorectal neoplasia. These studies are summarized below in Table 1. Adenomas and colorectal neoplasia were more prevalent in HBV-infected patients, particularly those who tested positive for HBsAg than in healthy controls [16,17]. Furthermore, Kim et al. substantially linked people with HBV DNA positivity to larger polyps and advanced adenomas, highlighting the part that active viral replication plays in promoting the development of premalignant lesions [18]. The correlation between HBsAg positive and a higher incidence of colorectal cancer and other solid tumors provided more evidence of HBV's systemic carcinogenic potential [19-21]. According to certain cohort studies, CHB patients, particularly those in younger populations, had an increased risk of extrahepatic malignancies and colorectal cancer (CRC), highlighting the significance of early screening in this community [17,22]. Liu et al. and Hong et al. confirmed the association between HBV infection and increased susceptibility to colorectal neoplasia, which includes both adenomas and colorectal cancer [23,24].

**Table 1:** Summary of studies showing the association of chronic hepatitis B and colonic neoplasia using global clinical data. CHB: chronic hepatitis B, HBV: hepatitis B virus, CRA: colorectal adenoma, DNA: deoxyribonucleic acid, HBsAg: hepatitis B surface antigen.

Name of the study	Authors	Year	Study Type	Results
Establishing the link between hepatitis B virus infection and colorectal adenoma	Patel BB, et al. [16]	2015	Retrospective case-control study in United States	Higher number of adenomas present in the distal colon in CHB patients as compared to healthy controls.
Chronic hepatitis infection is associated with extrahepatic cancer development: a nationwide population-based study in Taiwan	Kamiza AB, et al. [17]	2016	Cohort study involving Taiwanese population	CHB patients exhibited an increased risk of colorectal cancer and some other extrahepatic malignancies.
Hepatitis B virus infection is independently associated with advanced colorectal adenoma	Kim SH, et al. [18]	2018	Retrospective case-control study in South Korea	The HBV group had a higher rate of CRA, advanced adenoma, larger colorectal polyp size as compared to the uninfected group. This study revealed HBV DNA positivity is significantly associated with advanced colorectal adenoma formation(a premalignant lesion).
Hepatitis B and C rates are significantly increased in certain solid tumors: a large retrospective study	Kocoglu H, et al. [19]	2018	Retrospective case-control study in Turkey	HbsAg positivity ratio is significantly higher in solid organ tumors as compared to healthy controls.
Correlation between hepatitis B virus infection and colorectal neoplasia	Jung YS, et al. [20].	2019	Cross-sectional study in South Korea	The prevalence of colorectal neoplasia was higher in the HBsAg (+) than in HBsAg (-) participants.
Associations between hepatitis B virus infection and risk of all cancer types	Song C, et al. [21]	2019	Prospective cohort study in China	Individuals with HBsAg seropositivity were associated with increased risk of colorectal cancer and other solid organ malignancies.
Chronic hepatitis B virus infection associated with increased colorectal cancer risk in Taiwanese population	Su FH, et al. [22]	2020	Retrospective case-control study in Taiwan	Chronic HBV infection is significantly associated with an increased risk of colorectal cancer, particularly among younger patients
Chronic viral hepatitis is associated with colorectal neoplasia: a systematic review and meta-analysis	Hong SW, et al. [23]	2021	Systematic review and meta-analysis	Chronic viral hepatitis patients (both Hep B and Hep C) are associated with an increased risk of colorectal neoplasia, including both CRA and CRC.
Associations between hepatitis B virus infection and risk of colorectal cancer: a population-based prospective study	Liu T, et al. [24]	2021	Prospective cohort study in China	Significant positive association between HBV infection and the risk of incident CRC.

In summary, HBV infection is a significant risk factor for colorectal carcinogenesis in a range of populations around the world, from early premalignant lesions to late colorectal cancer. This is especially true when HBsAg positivity and elevated HBV DNA levels are present. These findings emphasize the importance of addressing HBV as a modifiable risk factor to detect and prevent colorectal cancer early. 

The connection between CRLM and HBV infection is more nuanced. According to one study, patients with CRC who were infected with HBV had a greater prevalence of CRLM, especially if they had synchronous metastases and were HBeAg positive, which may indicate that HBV plays a factor in early metastatic dissemination [25]. Conversely, a different study found that patients with HBV had a lower probability of metachronous CRLM, suggesting a possible preventive effect against delayed metastasis [26]. In support of this, some other studies observed that HBV infection reduces the risk of CRLM in colon cancer patients. However, it is linked to a higher incidence of extrahepatic metastases. Despite these differences in metastatic patterns, overall survival was unaffected by HBV infection [27]. According to a study by Wang et al., patients with HBV had better five-year survival rates and lower CRLM rates than non-infected individuals [28]. With specific reports of extended longevity for HBV-infected patients, chronic HBV infection was found to be an independent factor determining the development of CRLM [29,30]. Table 2 summarizes CHB and its association with CRLM.

**Table 2:** Summary of studies showing the association between chronic hepatitis B and colorectal liver metastasis in colon cancer patients. CRLM: colorectal liver metastasis, FIB-4: fibrosis-4 Index for Liver Fibrosis, APRI: aspartate aminotransferase-to-platelet ratio index, CHB: chronic hepatitis B, HBV: hepatitis B virus, HBeAg: hepatitis B envelope antigen.

Name of the study	Authors	Year	Type of study	Results
Active chronic hepatitis B increases the risk of colorectal liver metastasis - a retrospective cross-sectional study	Yang Y, et al. [25]	2021	Retrospective cross-sectional study in Asian population	A higher prevalence of CRLM noted in hepatitis B patients with colon cancer as compared to colon cancer alone. Additionally, those patients having HbeAg positivity had more prevalent synchronous CRLM.
Association between hepatitis B virus infection and colorectal liver metastasis: a meta-analysis	Liu R, et al. [26]	2021	Meta-analysis	HBV infection is associated with a significantly reduced risk of metachronous CRLM.
HBV infection decreases risk of liver metastasis in patients with colorectal cancer: a cohort study	Qiu HB, et al. [27]	2011	Case-control study	HBV infection significantly reduces the rate of CRLM in colon cancer patients. However, it is associated with a higher incidence of extrahepatic metastasis but does not impact the overall survival rates.
Colorectal liver metastases rarely occur in patients with chronic hepatitis virus infection	Wang FS, et al. [28]	2012	Retrospective cohort study	The HBV-infected group had significantly lower rates of CRLM, along with a higher 5-year survival rate as compared to the uninfected group.
Hepatitis B virus infection is an independent factor influencing the occurrence of liver metastasis in colorectal cancer: a retrospective analysis of 1413 cases	Qian HG, et al. [29]	2014	Retrospective cohort study	HBV infection is an independent factor for the occurrence of CRLM but does not affect survival rates.
Rare occurrence of metastatic colorectal cancers in livers with replicative hepatitis B infection	Song E, et al. [30]	2001	Retrospective cohort study	Chronic HBV infection has significantly lower rates of CRLM, thereby prolonging patient survival.
Effect of concomitant positive hepatitis B surface antigen on the risk of liver metastasis: a retrospective clinical study of 4033 consecutive cases of newly diagnosed colorectal cancer	Huo T, et al. [31]	2018	Retrospective cohort study	Concomitant CHB infection in colon cancer patients is associated with significantly increased risk of CRLM. Colon cancer patients who have HbsAg positivity have higher levels of FIB-4/APRI but less CRLM (which may be antimetastatic).

Moreover, HBsAg positivity was associated with a higher risk of CRLM, suggesting that this impact might be mediated by higher levels of APRI or FIB-4 [31]. In summary, CRLM risk and metastatic patterns are affected differently by synchronous and metachronous presentations of HBV infection, which may give some individuals a survival advantage. These findings highlight the complex relationship between HBV infection and metastatic colorectal cancer, which calls for more research into the potential prognostic and therapeutic effects of this association.

Discussion

**Figure 1:**
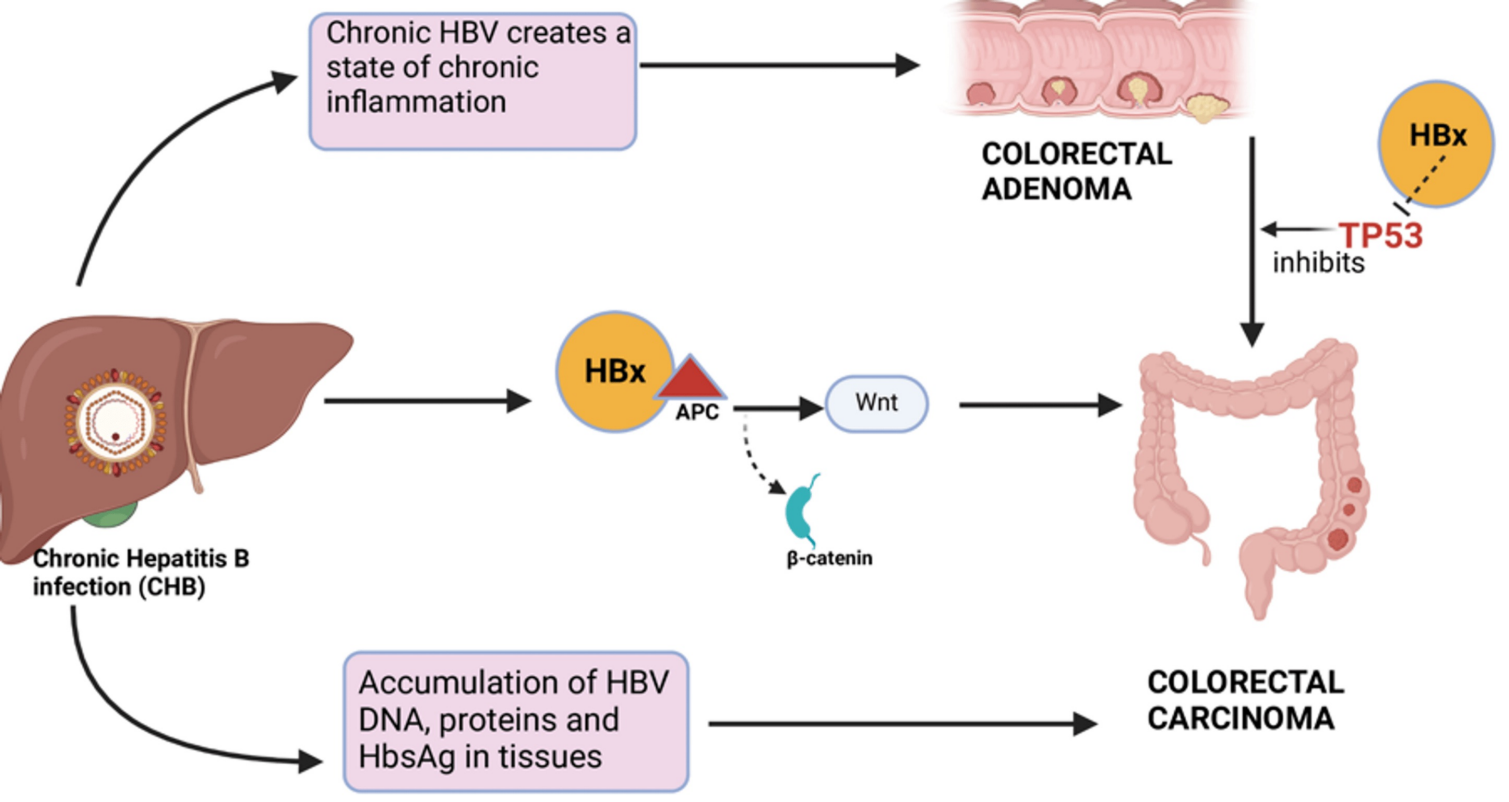
Mechanisms of hepatitis B virus (HBV) induced colorectal carcinogenesis. DNA: deoxyribonucleic acid, HBx: hepatitis B protein X, Wnt: wingless-related integration site, TP53: tumor protein 53, APC: adenomatous polyposis coli. Created using BioRender.com.

Multiple studies have linked CHB with an increased risk of CRC development in HBsAg-positive patients [19,20,22-24]. Additionally, some studies note an increased risk of other extrahepatic malignancies as well [17,21]. Besides the elevated risk of CRC, they have a higher risk of developing large-size colon polyps and advanced adenomas, mainly in the distal colon [16,18]. These findings point out the potential premalignant role of HBV. Our aim in this study is to explore all the potential pathophysiological mechanisms that are thought to contribute to these findings as depicted in Figure 1.

Viral Interaction With the TP53 Gene Potentiates the Adenoma-Carcinoma Sequence

HBV has been shown to accelerate the adenoma-carcinoma sequence, which leads to the progression of benign CRAs to malignant carcinomas. The adenoma-carcinoma sequence involves the stepwise accumulation of genetic mutations leading to a gradual transformation of colonic epithelial cells into malignant tumors. Initiated by APC (adenomatous polyposis coli) gene mutations, benign adenomatous polyps form, which may progress to advanced adenomas and invasive carcinomas, which are driven by mutations in genes such as KRAS, BRAF, and TP53, along with dysregulation of the Wnt signaling pathway [32,33]. The HBV genome contains the hepatitis B X protein (HBx), which has the ability to interact with the p53 tumor suppressor gene that plays an important role in the progression of CRAs to carcinomas [33]. This mechanism resembles the pathway through which hepatitis B induces hepatocellular carcinoma [34]. According to a study by Jung et al., HBV impacts the initial phases of this sequence more seriously than the advanced phases [20].

HBx Protein-Induced Activation of Wnt Signaling

HBx, after binding to the APC protein, can displace β-catenin from its degradation complex, causing activation of Wnt signaling and inducing malignant transformation [35].

Accumulation of the Viral Components in the Colon

Studies have shown that HBV can accumulate its viral DNA, protein, and HBsAg in tissues outside the liver, such as the colon, which creates a state of chronic inflammation in that area, possibly contributing to colonic carcinogenesis and extrahepatic tumorigenesis [36,37].

Modulation of Host Immune Response and Occult Hepatitis B Infection

HBV causes modulation of the host immune response, which may be responsible for extrahepatic tumorigenesis like CRC [38]. Occult hepatitis B is a condition that is negative for HBsAg but has HBV DNA in the serum [39]. These patients are known to have higher levels of interleukin-10 (IL-10), and interleukin-17 (IL-17), which are needed to clear viral infection, and lower levels of interleukin-12 (IL-12) and interferon gamma (IFN gamma) that are used to generate cellular immunity against viral infections, mainly HBV [40-42]. These immunomodulators increase the survival rate of HBV-infected cells and thus the presence of persistent infection.

Pre-existing Chronic Liver Disease

It has been noted that patients with liver cirrhosis, non-alcoholic fatty liver disease, or any other chronic liver disease tend to have increased rates of CRC [43-45]. Also, hepatitis B is a well-known risk factor for liver cirrhosis and hepatocellular cancer. There is a possibility that it can be an independent risk factor for CRC as well.

Shared Risk Factors and Regional Variations in Adenoma Prevalence

Hepatitis B infection and CRC share some common risk factors as well, such as age, smoking, male sex, alcohol intake, fatty liver, etc. [20]. Patel et al. noted some regional variation in adenoma prevalence with increased prevalence in the distal colon [16]. A possible reason can be that the chronic inflammatory state in CHB alters the distal colon, which gets affected more than the proximal colon due to differences in the gut microbiota [16,46]. A slower transit time in the distal colon can prolong the contact and might expose the mucosa to more carcinogenic substances present in the feces [47]. However, there is a possibility that this can be a screening bias because adenomas in the distal colon are more easily detected during colonoscopies than in the proximal colon.

Impact on CRLM

**Figure 2:**
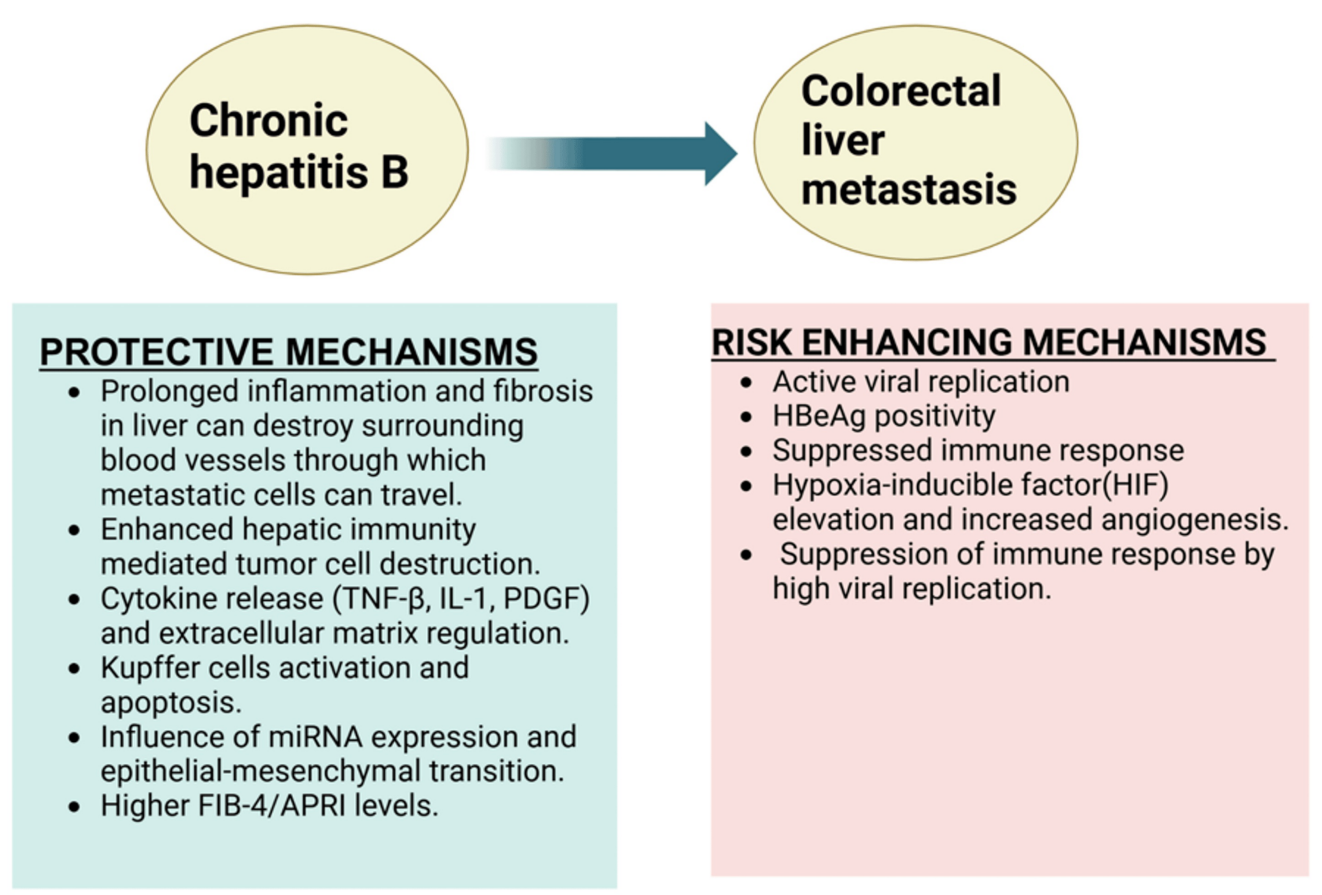
Dual role of hepatitis B infection in colorectal liver metastasis. TNF-β: tumor necrosis factor-β, IL-1: interleukin-1, PDGF: platelet-derived growth factor, miRNA: microribonucleic acid, FIB-4: fibrosis-4 index for liver fibrosis; APRI- aspartate aminotransferase-to-platelet ratio index, HBeAg: hepatitis B envelope. Created using BioRender.com.

The most common metastatic site for colon cancer is the liver due to its rich blood supply from the portal vein. Thus, the rate of CRLM significantly impacts the prognosis of these patients. HBV has been shown to have a dual nature when it comes to CRLM as shown in Figure 2. Some studies suggest a protective effect of HBV infection against CRLM, thereby prolonging patient survival [27,28,30]. This can be due to the fact that CHB creates a state of prolonged inflammation in the liver, leading to fibrosis and cirrhosis and eventually destroying the surrounding blood vessels through which the metastatic cells seed from the colon to the liver [48,49]. The release of various cytokines during inflammation of the liver, such as TNF-β, IL-1, and platelet-derived growth factor (PDGF), regulates the extracellular matrix and inhibits the metastasis of the tumor cells [50]. Additionally, the activation of Kupffer cells of the liver can cause fas-mediated apoptosis of the metastatic tumor cells [51]. This is not seen in HBV-induced primary HCC because persistent viral replication in CHB leads to immune tolerance and allows the already infected hepatocytes to escape destruction. In contrast, in metastatic cells, these immune evasion mechanisms are less developed and require time to establish. Some studies propose that hepatitis B viral replication enhances hepatic immunity, which destroys the tumor cells rather than any sort of structural changes in the liver [30,52,53]. It is also believed that HBV infection influences microRNA (miRNA) expression and the mesenchymal-epithelial transition of tumor cells, thereby inhibiting CRLM [54].

According to a study by Huo et al., the higher levels of FIB-4/APRI in HBsAg-positive colon cancer patients indicate significant liver fibrosis or cirrhosis but lower rates of CRLM [31]. However, there are some contradictory studies that revealed a complex relationship, indicating that concomitant CHB infection in colon cancer can elevate the risk of CRLM. In support of this, a higher prevalence of CRLM in HBV-infected patients with colon cancer is noted, particularly those with HBeAg positivity [25]. This can be due to the fact that HBx increased the levels of HIF (hypoxia-inducible factor), which can lead to the formation of new blood vessels in the liver, which can promote the spread and nourishment of metastatic cells [55]. Additionally, hepatitis B envelope antigen (HBeAg) positivity status means a high level of viral replication and active infection, which can suppress the immune system more effectively, reducing the body’s ability to eliminate metastatic cancer cells.

It is difficult to eliminate HBV DNA completely; however, the viral load and HBeAg elimination can be done using antiviral drugs. If a reduction in the levels of HBeAg can be achieved, it can reduce the chances of CRLM and improve patient survival.

Limitations

Our review focuses mainly on the pre-existing literature, which varies in study design and quality. Additionally, we utilized only two major databases, PubMed and Google Scholar, for our literature search. While these databases provide extensive coverage, some relevant studies available in other databases may not have been included. Since most of the studies are observational in nature, we can only establish an association rather than causation between CHB infection, CRC, and CRLM. These studies have been done globally and involve different patient populations, so variability in HBV genotypes and regional differences should be considered before forming any conclusion. Future research should include more large-scale prospective studies and clinical trials to confirm a cause-effect relationship and explore therapeutic implications.

## Conclusions

CHB infection has a dual impact on CRC and CRLM. While HBV infection increases the risk of advanced colorectal adenomas, it paradoxically protects against CRLM by inducing liver fibrosis, which alters the vascular environment and enhances hepatic immunity. Active HBV replication promotes liver angiogenesis and has a higher risk of synchronous CRLM. Over time, chronic inflammation leads to fibrosis and cirrhosis, reducing the likelihood of CRLM. Therefore, clinicians should consider both the pro- and antimetastatic effects of CHB while managing these patients. A comprehensive approach involving patient education on lifestyle modifications, hepatitis B vaccination, and strict antiviral adherence should be followed. Enhanced screening with frequent colonoscopies is recommended for early detection of precancerous lesions in CHB patients. Additionally, for CRC patients with HBV, strict antiviral control is important to prevent CRLM. Understanding these relationships is crucial to improving clinical outcomes and forming effective management strategies.
